# Silencing of ANGPTL8 Alleviates Insulin Resistance in Trophoblast Cells

**DOI:** 10.3389/fendo.2021.635321

**Published:** 2021-06-08

**Authors:** Yu Bai, Qiang Du, Le Zhang, Ling Li, Nana Wang, Bo Wu, Ping Li, Ling Li

**Affiliations:** Department of Endocrinology, Shengjing Hospital of China Medical University, Shenyang, China

**Keywords:** gestational diabetes mellitus, insulin resistance, trophoblast cells, angiopoietin like 8, c-Jun N-terminal kinase

## Abstract

This study aims to investigate the effect of angiopoietin like 8 (ANGPTL8) on gestational diabetes mellitus (GDM) and insulin resistance (IR). The GDM model was induced by high fat diet in mice, and IR was observed. The expression and secretion of ANGPTL8 were promoted in placenta of GDM mice. IR was induced in trophoblast cell HTR-8/SVneo by treatment of high concentration of insulin, and the expression levels of ANGPTL8 were increased. Silencing of ANGPTL8 alleviated IR and decreased glucose uptake in HTR-8/SVneo cells. However, the inflammation and oxidative stress in IR cells were not restrained by ANGPTL8 knockdown. In addition, c-Jun N-terminal kinase (JNK) signaling was activated by IR, which was inhibited by silencing of ANGPTL8. The effect of ANGPTL8 knockdown on IR was attenuated by JNK antagonist, and aggravated by JNK agonist, suggesting that ANGPTL8 affected IR by regulating JNK signaling. In conclusion, we demonstrated that the silencing of ANGPTL8 ameliorated IR by inhibiting JNK signaling in trophoblast cells. These findings may provide novel insights for diagnosis and treatment of GDM in clinic.

## Introduction

Gestational diabetes mellitus (GDM) is the most common metabolic disorder of pregnancy, defined as “the type of glucose intolerance that develops in the second and third trimester of pregnancy, resulting in hyperglycemia of variable severity” (1, 2). GDM is an increasing risk factor for severe pregnancy complications for both mother and child, including cesarean delivery, shoulder dystocia, macrosomia and neonatal hypoglycemia (3, 4). Women with GDM also have an increasing risk factor to develop type 2 diabetes mellitus (T2DM) and cardiovascular disease after pregnancy, and their offspring are at increasing risk for the development of obesity and T2DM in later life (5). The etiology of GDM remains unclear. However, pancreatic β-cell secretory impairment and insulin resistance (IR) are pivotal during pathogenesis of GDM (1).

IR is an impaired response to insulin that characterizes normal pregnancy. Physiologic IR results in increased insulin secretion. Women with GDM are unable to increase insulin production to compensate for the increased IR (6, 7). Pancreatic β-cell deterioration-induced IR and relative insulin deficiency are the primary metabolic changes in GDM. As β-cell function further declines, hyperglycemia becomes more severe. In addition, gluconeogenesis is increased as a result of IR and insulin deficiency. As a consequence, hyperglycemia is worsened (7). IR and insulin deficiency are often considered as therapeutic targets for GDM.

The expression of multiple genes involved in glucose and lipid metabolism are dysregulated in GDM. Angiopoietin like 8 (ANGPTL8) is a secretory protein, highly expressed in liver and fat, and it is involved in lipid metabolism (8, 9). ANGPTL8 has been reported to be increased in high fat diet (HFD)-induced diabetic mice, and the overexpression of ANGPTL8 alleviated hyperlipemia, glucose tolerance, IR and inflammation in diabetic mice (10). However, in another report, authors found that ANGPTL8 was highly expressed in omental fat of obese individuals with fatty and IR, and antisense oligonucleotide against *Angptl8* prevented hepatic IR in high-fat-fed rats (11). These studies suggested that the roles of ANGPTL8 in diabetes and IR remain uncertain. Its effect on GDM has not been investigated. Recently, ANGPTL8 was found higher in serum of women with GDM, compared with normal pregnancies (12), suggesting that ANGPTL8 may be involved in development of GDM.

In this study, we induced a GDM model *via* high fat diet (HFD) in mice. The effect of ANGPTL8 on GDM and IR was investigated.

## Material and Methods

### Animal Model

Healthy C57BL/6J male and female mice were kept in controlled environment (12 h/12 h light/dark cycles, 22 ± 1°C) with free access to food and water. After accommodation for one week, the female mice were randomly divided into two groups: HFD and normal fat diet (NFD). The mice in NFD group were fed with mouse standard chow purchased HUANYU BIO (Henan, China) including protein ≥18%, fat ≥4%, fiber ≤5%, ash ≤8%, carbohydrate ≤36%, calcium 1.0-1.8%, phosphorus 0.6-1.2%, and essential amino acid, vitamin and microelement. High fat chow was mixed with 15% lard oil, 10% yelk, 10% sugar and 65% standard chow. After dietary intervention for one week, breeding was conducted overnight in a 1:2 ratio; mating was confirmed by presence of a vagina mucous plug in the following morning, which represented gestation day (GD) 0.5. Oral glucose tolerance test (OGTT) was performed at GD0.5, 11.5 and 16.5. The weight of female mice was measured at GD0.5, 6.5, 12.5 and 18.5. The female mice were sacrificed at GD18.5, and the blood and placenta tissues were collected for subsequent detections.

The taken care of and conduct were line with Guide for the Care and Use of Laboratory Animals (8th, NIH), and the experimental procedure was approved by Ethical Committee of Shengjing Hospital of China Medical University.

### OGTT

After fasting overnight, the blood glucose of mice was detected. Then the mice were underwent gavage of glucose solution with 2 g/kg (400 mg/ml), and the blood glucose was determined after gavage for 30, 60 and 90 min.

### Homeostasis Model Assessment-Insulin Resistance (HOMA-IR)

After fasting overnight, the blood insulin was detected using an ELISA kit (USCN, Wuhan, Hubei, China) according to the manufacturer’s protocol. The serum was incubated with reagent A in ELISA plate well at 37°C for 1 h. Then the solution was removed, and reagent B was added to incubate for 30 min. After solution movement, TMB reagent was added into wells to react in the dark. About 10-20 minutes later, the reaction was stopped with stopping buffer. OD450 was determined with a microplate reader, and the insulin concentration was calculated according to the standard curve. The HOMA-IR value was calculated as follow:

HOMA-IR= blood glucose (mM)×blood insulin (mU/l)/22.5

### Detection of Lipid and Lipoprotein

The content of triglyceride (TG), total cholesterol (TC), high density lipoprotein cholesterol (HDL-C), low density lipoprotein cholesterol (LDL-C) in the serum was determined by kits (Jiancheng, Nanjing, Jiangsu, China) according to the manufacturer’s protocol.

### HE Staining

HE staining was performed to detect the histological changes in labyrinth zone of placenta tissue. The tissue was fixed with 4% paraformaldehyde overnight. After washing with flow water for 4 h, the tissue was dehydrated with grading concentration of ethanol (70% for 2 h, 80% overnight, 90% for 2 h and 100% for 1 h twice) and xylene for 30 min. Then the tissue was embedded with paraffin at 60°C for 2 h, and the paraffin mass was cut into sections of 5 μm, which were underwent deparaffinization with xylene for 15 min twice, 100% ethanol for 5 min twice, and 95%, 85%, 75% ethanol for 2 min, respectively. The sections were stained with hematoxylin (Solarbio, Beijing, China) for 5 min, soaked with 1% hydrochloric acid/ethanol for several seconds, and counterstained with eosin (Sangon, Shanghai, China) for 3 min. Finally, the sections were dehydrated again with grading concentration of ethanol (75% for 2 min, 85% for 2 min, 95% for 2 min, 100% for 5 min twice) and xylene (for 10 min twice), mounted with gum and observed with a microscope (Olympus, Tokyo, Japan) at 200× magnification.

### Periodic Acid Schiff (PAS) Staining

PAS staining was used for detection of glycogen content in labyrinth zone of placenta tissue. The tissue was made into paraffin sections as previous description, and deparaffinization was performed with xylene and ethanol. The sections were incubated with periodic acid solution (Leagene, Beijing, China) for 10 min, washed with water for 5 min, stained with schiff buffer (Leagene) for 15 min, washed with water for 5 min, and countered with hematoxylin for 2 min. After dehydration with ethanol and xylene, the sections were mounted with gum and photographed with a microscope at 200× magnification.

### Real-Time PCR

The total RNA was extracted with RNApure extraction kit (BioTeke, Beijing, China), and the concentration was measured with NANO 2000 ultraviolet spectrophotometer (Thermo Scientific, Waltham, MA, USA). The RNA was reversely transcribed into cDNA with M-MLV reverse transcription (TAKARA, Japan) with Oligo(dT) and random primer. The instruments and reagents used in reverse transcription were RNase-free. The cDNA was used for real-time PCR with Taq HS Perfect Mix (TAKARA) and SYBR Green (BioTeke) to detect the mRNA levels of ANGPTL8, tumor necrosis factor-α (TNF-α), interleukin (IL)-1β and IL-6. β-actin served as the internal control. The PCR procedure was set as follow: 94°C for 5 min 20 s, 60°C for 30 s, 72°C for 40 s, and 40 cycles of 72°C for 5 min 30 s, 40°C for 4 min 30 s, melting 60-90°C every 1°C for 1 s, and incubated at 25°C for several minutes. The PCR ran in Exicycler TM96 quantitative thermal block. The data were calculated using 2^-ΔΔCt^ method. The primers were purchased from Genscript (Nanjing, Jiangsu, China), and the information was shown in **Table 1**.

**Table 1:** The information of primers in this study.

Name	Sequence (5’-3’)
Mus ANGPTL8 F	CGGTCAAGCCCACCAAGA
Mus ANGPTL8 R	TGCTGCTCTGCCATCTCCC
Mus β-actin F	CTGTGCCCATCTACGAGGGCTAT
Mus β-actin R	TTTGATGTCACGCACGATTTCC
Homo ANGPTL8 F	CTTCGGGCAAGCCTGTT
Homo ANGPTL8 R	GCTGTCCCGTAGCACCTTC
Homo IL-1β F	GAATCTCCGACCACCACTAC
Homo IL-1β R	CACATAAGCCTCGTTATCCC
Homo IL-6 F	TGCCTTCCCTGCCCCAGT
Homo IL-6 R	GTGCCTCTTTGCTGCTTTCA
Homo TNF-α F	CGAGTGACAAGCCTGTAGCC
Homo TNF-α R	TTGAAGAGGACCTGGGAGTAG
Homo β-actin F	CACTGTGCCCATCTACGAGG
Homo β-actin R	TAATGTCACGCACGATTTCC

### Western Blot

The protein was extracted with RIPA lysis buffer (Beyotime, Haimen, Jiangsu, China) on ice, and the concentration was determined with BCA protein concentration kit according to the manufacturer’s protocol. After denaturation in boiling for 5 min, equal amount of protein was separated with SDS-PAGE, and the concentration of polyacrylamide gel varied from 5% to 12% according to the protein size. After electrophoresis for about 2.5 h, the protein was transferred onto PVDF membrane (Thermo Scientific). After blocking with skim milk at room temperature for 1 h, the membrane was incubated with one of the following antibodies at 4°C overnight: rabbit anti-ANGPTL8 (1:1000; cat. no. A18133, ABclonal, Wuhan, Hubei, China), rabbit anti-insulin receptor β (IRβ) (1:1000; cat. no. AF6099, Affinity, Changzhou, Jiangsu, China), rabbit anti-p-IRβ(Tyr1361) (1:1000; cat. no. AF3099, Affinity), rabbit anti-insulin receptor substrate-1 (IRS-1) (1:1000; cat. no. AF6273, Affinity), rabbit anti-p-IRS-1(Ser307) (1:1000; cat. no. AF3272, Affinity), rabbit anti-p-IRS1(Tyr895) (1:1000; cat. no. 3070, CST, Boston, MA, USA), rabbit anti-Akt (1:1000; cat. no. 4685, CST), rabbit anti-p-Akt(Ser473) (1:1000; cat. no. 4060, CST), rabbit anti-glucose transporter (GLUT)1 (1:1000; cat. no. AF0173, Affinity), mouse anti-GLUT4 (1:1000; cat. no. BF1001, Affinity), rabbit anti-Rho associated coiled-coil containing protein kinase (ROCK)I (1:2000; cat. no. 21850-1-AP, Proteintech, Wuhan, Hubei, China), rabbit anti-ROCKII (1:2000; cat. no. 21645-1-AP, Proteintech), rabbit anti-MYPT1 (1:1000; cat. no. AF5444, Affinity), rabbit anti-p-MYTP1(Thr853) (1:1000; cat. no. AF5445, Affinity), rabbit anti-c-Jun N-terminal kinase (JNK) (1:500; cat. no. AF6318, Affinity), rabbit anti-p-JNK(Thr183/Tyr185) (1:500; cat. no. AF3318, Affinity) and mouse anti-β-actin (1:2000; cat. no. 60008-1-Ig, Proteintech). After rinsing with TBST, the membrane was incubated with goat anti-mouse or rabbit secondary labeled with HRP (1:10000; Proteintech) at 37°C for 40 min. Finally, the membrane was reacted with ECL reagent for 5 min, followed with signal exposure in the dark. The optical density of the bands was analyzed with Gel-Pro-Analyzer software. β-actin served as the internal control.

### Cell Culture and Treatment

Immortalized human chorionic trophoblast line HTR-8/SVneo was purchased from Procell (Wuhan, Hubei, China), and cultured in RPMI-1640 medium supplemented with 5% fetal bovine serum (FBS) (Biological industries, Kibbutz Beit-Haemek, Israel) at 37°C with 5% CO_2_.

The transfection was performed using Lipofectamine 2000 (Invitrogen, Carlsbad, CA, USA) with serum-free medium.

The cells were incubated with insulin (Solarbio) of 10^-6^ mol/l for 48 h to induce IR, and insulin of 10^-7^ mol/l was used to stimulate cells to verify the IR.

JNK signaling agonist Anisomycin (1 μg/ml) (Yuanye, Shanghai, China) and antagonist SP600125 (20 μM) (Aladdin, Shanghai, China) was used to treat cells for 30 min to activate or inactivate the JNK signaling.

### Immunofluorescent Staining

Immunofluorescent staining was used to detect the expression and distribution of GLUT1 and GLUT4. The cells were pre-seeded on glass slides, and fixed with 4% paraformaldehyde for 15 min. After washing with PBS to remove the residual paraformaldehyde, the cells were permeated with 0.1% TritonX-100 for 30 min to permeate cytomembrane. Then the cells were blocked with goat serum at room temperature for 15 min to block the non-specific antigens, and incubated with antibody against GLUT1 or GLUT4 (1:200; cat. no. AF0173, BF1001, Affinity) at 4°C overnight. After washing with PBS, the cells were incubated with Cy3-labeled secondary body for 60 min in the dark, and incubated with DAPI (Aladdin) to stain nuclei. Finally, the cells were mounted with anti-fading reagent, and photographed with a fluorescence microscope (Olympus) at 400× magnificance.

### ELISA

The ANGPTL8 content in serum was detected with an ELISA kit (USCN) according to manufacturer’s protocol. First, the serum sample or cell supernatant was incubated in the ELISA plate well at 37°C for 1 h. Then the solution was removed, and reagent A was added into the ELISA plate well to incubate for 1 h. Subsequently, the well was washed with washing buffer, and incubated with reagent B for 1 h. Finally, TMB substrate solution was added into the well to react for 20 min in the dark, and OD450 was measured. The concentration was calculated according to standard curve.

The content of inflammatory factors, TNF-α, IL-1β and IL-6 in serum were determined with ELISA kits (USCN) according to the manufacturer’s introductions. The detail steps were similar to above description.

### Detection of Reactive Oxygen Species (ROS)

The content of ROS in cells was detected with an ROS assay kit (Beyotime). The cells were collected and washed with PBS. The cells were incubated with DCFH-DA solution (1:1000 diluted with serum-free medium) at 37°C for 20 min, and the reaction system was mixed upside-down every 3 min. Then the cells were washed with PBS to remove the free DCFH-DA, and fluorescence was detected by flow cytometer (ACEA, San Diego, USA).

### Detection of Superoxide Dismutase (SOD) Activity

The SOD activity in the cells was detected by kit (Jiancheng) according to the manufacturer’s protocol. The cells were lysed with ultrasound, and the total protein was extracted. The protein concentration was determined with BCA kit, and standard curve was drawn. The protein sample was incubated with color developing agent, and OD550 was measured.

SOD activity (U/mgprot) = ((contro OD550 - sample OD550)/control OD550)/50% × (volume of reaction solution (ml)/sample volume (ml)/protein sample volume (ml))

### Statistical Analysis

The data in this study were present as mean ± SD in six (*in vivo*) or three individuals (*in vitro*). The data from animal experiments were analyzed with Student’s t test, and data from cell experiments were analyzed with one-way or two-way analysis of variance followed with Bonferroni post-hoc test. A p value less than 0.05 was considered as statistically significant (*p<0.05, **p<0.01, ***p<0.001, ns, no significance).

## Results

### ANGPTL8 Was Increased in Serum and Placenta Tissues of GDM Mice

**Figure 1:**
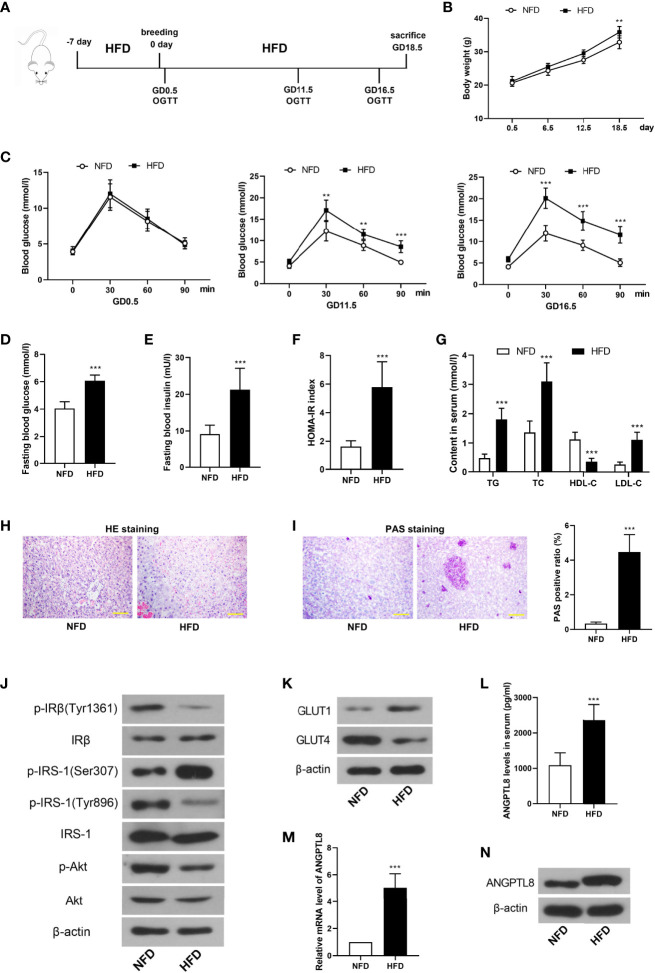
Angiopoietin like-8 (ANGPTL8) was increased in serum and placenta tissues of gestational diabetes mellitus (GDM) mice. **(A)** The mice were treated as described in the chart. **(B)** The body weight of mice in normal fat diet (NFD) and high fat diet (HFD)**** groups. **(C)** Oral glucose tolerance test (OGTT) was performed at gestational day (GD)0.5, 11.5 and 16.5. **(D, E)** Fasting blood glucose and insulin levels were measured at GD18.5. **(F)** Homeostasis model assessment insulin resistance (HOMA-IR) was calculated as follow: HOMA-IR= blood glucose (mM)×blood insulin (mU/l)/22.5. **(G)** The contents of triglyceride (TG), total cholesterol (TC), high density lipoprotein (HDL-C) and low density lipoprotein (LDL-C) in serum were detected. **(H)** HE staining was performed to detect the pathological changes in labyrinth zone of placenta tissues. **(I)** Periodic acid Schiff (PAS) staining was carried out to detect the glycogen accumulation in labyrinth zone of placenta tissues. **(J)** Western blot was used to determine the levels of insulin signaling related molecules, p-IRβ(Tyr1361), IRβ, p-IRS-1(Ser307), p-IRS-1(Tyr896), IRS-1, p-Akt and Akt in placenta tissues. **(K)** The expression levels of glucose transporter 1 (GLUT1) and GLUT4 in placenta tissues. **(L)** The serum level of ANGPTL8 in mice. **(M, N)** The mRNA and protein levels of ANGPTL8 in placenta tissues. (the scale bar represents 100 μm; **p < 0.01, ***p < 0.001 vs. NFD).

GDM was induced by HFD in mice, and the treatments were described in the **Figure 1A**. HFD induced more significant weight gain than NFD during pregnancy (**Figure 1B**). **Figure 1C** revealed that HFD led to glucose tolerance at GD11.5 and GD16.5. The fasting blood glucose and insulin levels were detected at GD18.5. The results showed that HFD caused significant elevation of fasting blood and insulin levels and HOMA-IR index in pregnant mice (**Figures 1D–F**). In addition, the content of triglyceride, cholesterol and low density lipoprotein in serum was increased and that of high density lipoprotein was decreased in HFD mice (**Figure 1G**).

Next, HE and PAS staining revealed that HFD induced inflammatory infiltration and glycogen accumulation in placenta tissues of pregnant mice (**Figures 1H, I**). Then insulin signaling-related molecules were detected. As shown in **Figure 1J**, the phosphorylation of IRβ(Tyr1361), IRS-1(Tyr896) and Akt was decreased, and that of IRS-1(Ser307) was increased in placenta of HFD mice, which suggested the deactivation of insulin signaling. However, the previous results showed higher blood insulin level in HFD. These results demonstrated the occurrence of IR in placenta of HFD mice. The expression level of a glucose transporter GLUT1 was increased, while that of another glucose transporter GLUT4 was decreased in placenta (**Figure 1K**). In addition, ANGPTL8 was detected. The results showed that the content of ANGTPL8 in serum was increased, and its mRNA and protein levels in placenta were also increased in HFD mice (**Figures 1L–N**).

### Silencing of ANGPTL8 Inhibited IR in Trophoblast Cells

**Figure 2:**
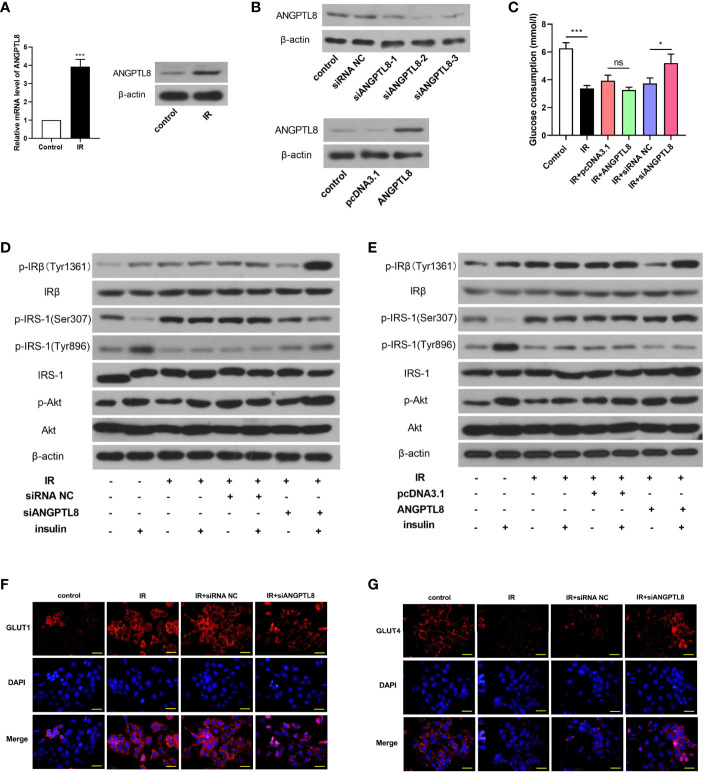
Silencing of ANGPTL8 inhibited IR in trophoblast cells. **(A)** The mRNA and protein levels of ANGPTL8 in HTR-8/SVneo cells with IR. **(B)** The expression levels of ANGTPL8 were detected by western blot after overexpression or knockdown of ANGPTL8 in HTR-8/SVneo cells. **(C)** The glucose consumption in HTR-8/SVneo cells with overexpression or silence of ANGPTL8 was measured. **(D, E)** The levels of insulin signal related molecules, p-IRβ(Tyr1361), IRβ, p-IRS-1(Ser307), p-IRS-1(Tyr896), IRS-1, p-Akt and Akt in HTR-8/SVneo cells with IR, overexpression or knockdown of ANGPTL8 or/and insulin stimulation. **(F, G)** Immunofluorescent staining was used to detect the expression and distribution of GLUT1 and GLUT4 in HTR-8/SVneo cells. (the scale bar represents 50 μm; *p < 0.05, ***p < 0.001, ns, no significance).

In order to investigate the role of ANGPTL8 in IR during pregnancy, IR was induced by high concentration of insulin treatment (10^-6^ mol/l) in HTR-8/SVneo cells. Western blot results showed that ANGPTL8 was increased in trophoblast cells with IR (**Figure 2A**). Then ANGPTL8 was silenced with interference RNAs, and ectopic expressed with overexpression plasmid. The effectiveness of knockdown or overexpression was confirmed with western blot (**Figure 2B**). To investigate the effect of ANGPTL8 on IR, glucose consumption was measured. As shown in **Figure 2C**, the treatment of high concentration of insulin induced obvious IR in trophoblast cells, which was significantly alleviated by ANGPTL8 knockdown. However, the effect of ANGPTL8 overexpression on IR was mild (**Figure 2C**). Thereafter, the insulin signaling was examined. As shown in **Figures 2D, E**, the insulin signaling was rapidly activated by physiological concentration of insulin (10^-7^ mol/l) in untreated cells, evidenced by increased levels of p-IRβ(Tyr1361), p-IRS-1(Tyr896) and p-Akt, and decreased level of p-IRS-1(Ser307). After IR induction, the cells did not respond to physiological concentration of insulin, and the levels of insulin signaling related molecules almost unchanged. The IR was effectively ameliorated in ANGPTL8-silenced cells, evidenced by the enhanced response to physiological concentration of insulin (**Figure 2D**). However, the overexpression of ANGPTL8 did not affect IR in trophoblast cells (**Figure 2E**). Immunofluorescent staining revealed that IR induced increase of GLUT1 and decrease of GLUT4, which was consistence with those results *in vivo*. After silencing of ANGPTL8, the expression of GLUT1 was reduced, and that of GLUT4 was elevated (**Figures 2F**, **G**), suggesting the enhanced glucose consumption.

### ANGPTL8 Activated JNK and ROCK Signaling

**Figure 3:**
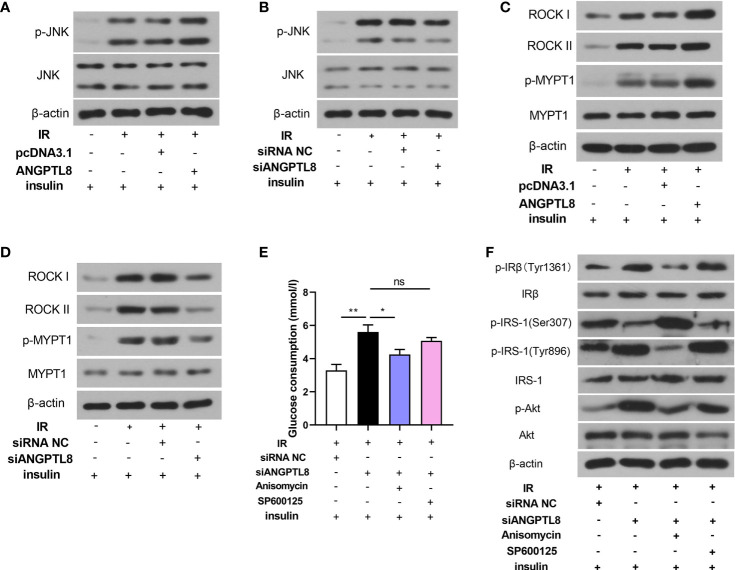
ANGPTL8 activated JNK and ROCK signaling. **(A, B)** The levels of p-JNK and JNK in HTR-8/SVneo cells with IR, overexpression or silence of ANGPTL8 or/and insulin stimulation. **(C, D)** The levels of ROCK I, ROCK II, p-MYPT1 and MYPT1 in HTR-8/SVneo cells. **(E)** Glucose consumption was determined in HTR-8/SVneo cells with ANGPTL8 knockdown, JNK agonist Anisomycin (1 μg/ml) or JNK antagonist SP600125 (20 μM), and insulin stimulation. **(F)** The levels of insulin signaling related molecules were determined in HTR-8/SVneo cells. (*p < 0.05, **p < 0.01, ns. no significance).

Since the important roles of JNK and ROCK signaling in diabetes mellitus, molecules in these two signaling pathways were detected. As shown in **Figure 3**, both JNK and ROCK signaling were activated in trophoblast cells with IR, evidenced by increased levels of p-JNK and p-MYPT1. The expression levels of ROCKI and ROCKII were also increased after IR induction, which may promote the activity of ROCK signaling (**Figures 3C, D**). After ANGPTL8 overexpression, the activity of JNK and ROCK was further increased, and it was declined after ANGPTL8 knockdown (**Figures 3A–D**). To explore the detail mechanism of ANGPTL8 function, a JNK signaling agonist Anisomycin and a JNK signaling antagonist SP600125 were used in trophoblast cells. As shown in **Figure 3E**, silencing of ANGPTL8 significantly restrained IR-induced glucose uptake obstruction. The effect of ANGPTL8 knockdown was attenuated by Anisomycin, but strengthened by SP600125 (**Figure 3E**). In addition, the insulin signaling was determined. As shown in **Figure 3F**, the mitigation effect of siANGPTL8 on IR was relieved by Anisomycin, but strengthened by SP600125, evidenced by the changes of insulin signaling related molecules. The results in this section suggested that silencing of ANGPTL8 restrained IR by inhibiting JNK signaling in trophoblast cells.

### Silencing of ANGPTL8 did not Ameliorate Inflammation and Oxidative Stress in HTR-8/SVneo Cells With IR

**Figure 4:**
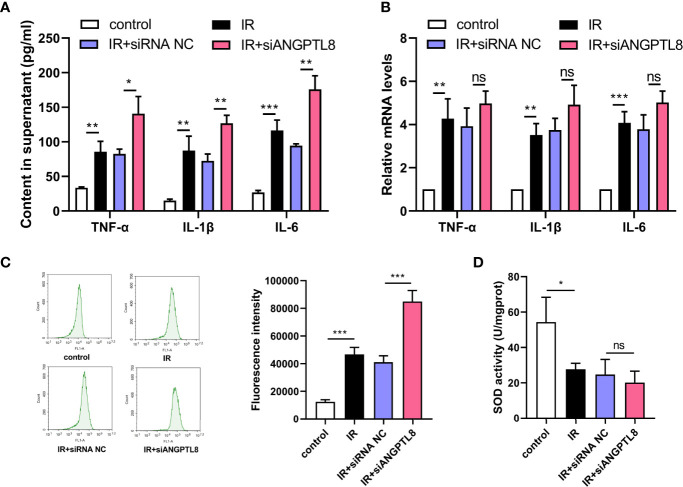
Silencing of ANGPTL8 did not ameliorate inflammation and oxidative stress in HTR-8/SVneo cells with IR. **(A)** The levels of TNF-α, IL-1β and IL-6 in supernatant of HTR-8/SVneo cells with IR and ANGPTL8 knockdown. **(B)** The mRNA levels of TNF-α, IL-1β and IL-6 in HTR-8/SVneo cells with IR and ANGPTL8 knockdown. **(C)** ROS content in HTR-8/SVneo cells was measured with DCFH-DA staining. **(D)** The SOD activity in HTR-8/SVneo cells. (*p < 0.05, **p < 0.01, ***p < 0.001, ns, no significance).

Inflammation and oxidative stress response in placenta play crucial roles in GDM, so inflammation and oxidative stress were detected in trophoblast cells after IR induction. From the data shown in **Figure 4**, we found that the expression and secretion of IL-1β, IL-6 and TNF-α were all increased in trophoblast cells with IR. The inflammatory response induced by IR was not attenuated by silencing of ANGPTL8 (**Figures 4A, B**). IR also led to ROS production and decrease of SOD activity, suggesting oxidative stress response. After silencing of ANGPTL8, the ROS content was further increased, and the SOD activity did not changed (**Figures 4C, D**). The results in this section suggested that ANGPTL8 knockdown did not alleviated inflammation and oxidative stress response in trophoblast cells with IR.

## Discussion

Insulin is synthesized and secreted in pancreatic β cells, which cooperates with glucagon to maintain plasma glucose homeostasis. Insulin signaling is initiated with insulin binding to the insulin receptor, which is internalized and phosphorylated. The phosphorylated insulin receptor activated IRS family of proteins, and PI3K/Akt signaling was subsequently activated (13, 14). Impaired insulin receptor activities lead to IR, the key factor in the pathology of metabolic disorders including diabetes (15).

During pregnancy, the mother’s body undergoes a series of physiological changes in order to support the demands of the growing fetus, and one important metabolic adaptation is insulin sensitivity. During early gestation, insulin sensitivity increases, promoting the uptake of glucose into adipose stores in preparation for the energy demands of later pregnancy (16). However, as pregnancy progresses, a surge of local and placenta hormones together promote a stage of IR (17). As a result, blood glucose is slightly elevated, and this glucose is readily transported across the placenta to fuel the growth of the fetus (18). In order to maintain glucose homeostasis, pregnant women compensate for these changes through hypertrophy and hyperplasia of pancreatic β cells, as well as increased glucose-stimulated insulin secretion (19). Moreover, the maternal insulin sensitivity returns to pre-pregnancy levels within a few days of delivery (20). For some reasons, the normal metabolic adaptations to pregnancy do not adequately occur in all pregnancies, resulting in GDM.

In our study, HFD induced GDM in pregnancy mice, shown as hyperglycemia, hyperinsulinemia, and pathological IR in placenta. The transcription, translation and secretion of ANGPTL8 were enhanced in GDM mice. At the same time, IR was induced in HTR-8/SVneo cells by treatment of high concentration of insulin (10^-6^ mol/l) *in vitro*, and the expression of ANGPTL8 was also increased. Silencing of ANGPTL8 significantly restrained IR in trophoblast cells, while the effect of ANGPTL8 overexpression on IR was negligible. We hypothesized that ANGPTL8 may be essential for IR, but it functioned independently of dosage. The roles of ANGPTL8 in T2DM are contradictory in previous reports (10, 11). We firstly investigated its role in GDM, and demonstrated that silencing of ANGPTL8 alleviated IR in trophoblast cells. However, its effect on GDM mice needs to be further studied by experiments *in vivo*.

Excess glycogen accumulation was found in placenta of GDM mice, suggesting abnormal glucose uptake and gluconeogenesis. At the same time, the expression of GLUT1 was increased, while that of GLUT4 was decreased in placenta. GLUT1 is highly expressed in placenta, and the glucose uptake in placenta is performed mainly *via* GLUT1. GLUT1 acts independently of insulin, and it is usually activated in placenta with IR (21). GLUT1 was reported highly expressed in placenta of women with GDM, especially those with macrosomia offspring (22). Macrosomia is a common complication of GDM, may be result from excessive uptake of glucose and lipid. We hypothesized that the increased expression of GLUT1 may lead to excess glucose uptake and gluconeogenesis in placenta. However, the expression of GLUT4 was decreased in GDM mice. GLUT4 was mainly expressed in fat and muscle. It is an insulin-responsive glucose transport (23). GLUT4 was low expressed in adipocytes of experimental diabetic rats with IR (24). It was also found to be decreased in placentas of women with GDM, and the decrease was most prominent in women receiving insulin (25). This may be result from inactivation of insulin signaling. In trophoblast cells with IR induction, the expression of GLUT1 was increased and that of GLUT4 was decreased. Moreover, the GLUT4 could not respond to insulin stimulation in cells with IR. These were similar with results in mice. However, the glucose uptake was reduced in IR cells, which was inconsistent with the excess glycogen accumulation *in vivo*. We hypothesized that GLUT4 may be more abundantly distributed in HTR-8/SVneo cells, compared with placenta tissue. The glucose uptake of HTR-8/SVneo cells may be mainly performed by GLUT4, the insulin-responsive transporter. Our hypothesis was supported by the subsequent results, which showed that after silencing of ANGPTL8, the IR was restrained, accompanied with the promoted expression of GLUT4 and recovery of glucose uptake. Certainly, this hypothesis needs more experiments to confirm.

JNK signaling has been reported to be activated in STZ-induced diabetic rats (26), and JNK inhibitor alleviated 2,3,7,8-tetrachlorodibenzo-p-dioxin (TCDD)-induced IR in adipocytes (27). In our study, JNK agonist aggravated IR induced by high concentration of insulin treatment, and JNK antagonist attenuated IR in trophoblast cells. The effect of ANGPTL8 knockdown was abolished by JNK antagonist. So we deduced that silencing of ANGPTL8 ameliorated IR by suppressing JNK signaling in trophoblast cells. ROCK signaling was also activated in diabetic rats (26), and a ROCK inhibitor, fasudil, has been demonstrated to retrained experimental IR in trophoblast cells in our another paper (28).

In addition, IR is often accompanied with inflammation in diabetic patients or animals. In our study, the inflammatory reaction was observed in insulin resistant cells, accompanied with the oxidative stress response. After silencing of ANGPLT8, the inflammation and oxidative stress were not inhibited, which were not consistent with results of IR. Although IR and inflammation often occur at the same time, their mechanisms are not exactly the same. In our study, the silencing of ANGPTL8 alleviated IR but not inflammation in trophoblast cells. Its effect on GDM animal *in vivo* still needs to be further studied by more experiments.

In conclusion, we demonstrated that ANGPTL8 was highly expressed in placenta of GDM mice and IR trophoblast cells. The silencing of ANGPTL8 alleviated high concentration of insulin treatment-induced IR by inhibiting JNK signaling. But the inflammation in IR trophoblast cells was not restrained. These results may provide novel insights for diagnosis and treatment of GDM in clinic.

## Data Availability Statement

The original contributions presented in the study are included in the article/supplementary material. Further inquiries can be directed to the corresponding author.

## Ethics Statement

The animal study was reviewed and approved by Ethical Committee of Shengjing Hospital of China Medical University.

## Author Contribution

YB and LL (8th author) designed the project. YB, QD, LZ, LL (4th author), NW, BW, and PL performed the experimental and analyzed the data. YB drafted the manuscript. All authors contributed to the article and approved the submitted version.

## Funding

This study was supported by grants from the 345 talent project plan of Shengjing Hospital of China Medical University and the Department of Education Foundation of Liaoning Province [No. L2015568].

## Conflict of Interest

The authors declare that the research was conducted in the absence of any commercial or financial relationships that could be construed as a potential conflict of interest.

## References

[1] Chiefari E Arcidiacono B Foti D Brunetti A. Gestational Diabetes Mellitus: An Updated Overview. J Endocrinol Invest (2017) 40(9):899–909. 10.1007/s40618-016-0607-5 28283913

[2] American Diabetes A. 2. Classification and Diagnosis of Diabetes. Diabetes Care (2016) 39:S13–22. 10.2337/dc16-S005 26696675

[3] Bellamy L Casas JP Hingorani AD Williams D. Type 2 Diabetes Mellitus After Gestational Diabetes: A Systematic Review and Meta-Analysis. Lancet (2009) 373(9677):1773–9. 10.1016/S0140-6736(09)60731-5 19465232

[4] Sullivan SD Umans JG Ratner R. Gestational Diabetes: Implications for Cardiovascular Health. Curr Diabetes Rep (2012) 12(1):43–52. 10.1007/s11892-011-0238-3 22037824

[5] Group HSCR. The Hyperglycemia and Adverse Pregnancy Outcome (Hapo) Study. Int J Gynaecol Obstet (2002) 78(1):69–77. 10.1016/S0020-7292(02)00092-9 12113977

[6] Reece EA Leguizamon G Wiznitzer A. Gestational Diabetes: The Need for a Common Ground. Lancet (2009) 373(9677):1789–97. 10.1016/S0140-6736(09)60515-8 19465234

[7] Harlev A Wiznitzer A. New Insights on Glucose Pathophysiology in Gestational Diabetes and Insulin Resistance. Curr Diabetes Rep (2010) 10(3):242–7. 10.1007/s11892-010-0113-7 20425589

[8] Ren G Kim JY Smas CM. Identification of RIFL, a Novel Adipocyte-Enriched Insulin Target Gene With a Role in Lipid Metabolism. Am J Physiol Endocrinol Metab (2012) 303(3):E334–51. 10.1152/ajpendo.00084.2012 PMC3423120 22569073

[9] Zhang R. Lipasin, a Novel Nutritionally-Regulated Liver-Enriched Factor That Regulates Serum Triglyceride Levels. Biochem Biophys Res Commun (2012) 424(4):786–92. 10.1016/j.bbrc.2012.07.038 22809513

[10] Luo D Chen X Yang W Ran W Wen Z. Angiopoietin-Like 8 Improves Insulin Resistance and Attenuates Adipose Tissue Inflammation in Diet-Induced Obese Mice. Exp Clin Endocrinol Diabetes (2020) 128(5):290–6. 10.1055/a-0725-7897 30257264

[11] Vatner DF Goedeke L Camporez JG Lyu K Nasiri AR Zhang D. Angptl8 Antisense Oligonucleotide Improves Adipose Lipid Metabolism and Prevents Diet-Induced NAFLD and Hepatic Insulin Resistance in Rodents. Diabetologia (2018) 61(6):1435–46. 10.1007/s00125-018-4579-1 PMC5940564 29497783

[12] Huang Y Chen X Chen X Feng Y Guo H Li S. Angiopoietin-Like Protein 8 in Early Pregnancy Improves the Prediction of Gestational Diabetes. Diabetologia (2018) 61(3):574–80. 10.1007/s00125-017-4505-y 29167926

[13] Hall C Yu H Choi E. Insulin Receptor Endocytosis in the Pathophysiology of Insulin Resistance. Exp Mol Med (2020) 52(6):911–20. 10.1038/s12276-020-0456-3 PMC7338473 32576931

[14] Kulkarni RN. Receptors for Insulin and Insulin-Like Growth Factor-1 and Insulin Receptor Substrate-1 Mediate Pathways That Regulate Islet Function. Biochem Soc Trans (2002) 30(2):317–22. 10.1042/bst0300317 12023872

[15] Chen Y Huang L Qi X Chen C. Insulin Receptor Trafficking: Consequences for Insulin Sensitivity and Diabetes. Int J Mol Sci (2019) 20(20):5007. 10.3390/ijms20205007 PMC6834171 31658625

[16] Di Cianni G Miccoli R Volpe L Lencioni C Del Prato S. Intermediate Metabolism in Normal Pregnancy and in Gestational Diabetes. Diabetes Metab Res Rev (2003) 19(4):259–70. 10.1002/dmrr.390 12879403

[17] Catalano PM Tyzbir ED Roman NM Amini SB Sims EA. Longitudinal Changes in Insulin Release and Insulin Resistance in Nonobese Pregnant Women. Am J Obstet Gynecol (1991) 165(6 Pt 1):1667–72. 10.1016/0002-9378(91)90012-G 1750458

[18] Phelps RL Metzger BE Freinkel N. Carbohydrate Metabolism in Pregnancy. XVII. Diurnal Profiles of Plasma Glucose, Insulin, Free Fatty Acids, Triglycerides, Cholesterol, and Individual Amino Acids in Late Normal Pregnancy. Am J Obstet Gynecol (1981) 140(7):730–6. 10.1016/0002-9378(81)90731-6 7020420

[19] Parsons JA Brelje TC Sorenson RL. Adaptation of Islets of Langerhans to Pregnancy: Increased Islet Cell Proliferation and Insulin Secretion Correlates With the Onset of Placental Lactogen Secretion. Endocrinology (1992) 130(3):1459–66. 10.1210/en.130.3.1459 1537300

[20] Plows JF Stanley JL Baker PN Reynolds CM Vickers MH. The Pathophysiology of Gestational Diabetes Mellitus. Int J Mol Sci (2018) 19(11):3342. 10.3390/ijms19113342 PMC6274679 30373146

[21] Ruiz-Palacios M Ruiz-Alcaraz AJ Sanchez-Campillo M Larque E. Role of Insulin in Placental Transport of Nutrients in Gestational Diabetes Mellitus. Ann Nutr Metab (2017) 70(1):16–25. 10.1159/000455904 28110332

[22] Yao G Zhang Y Wang D Yang R Sang H Han L. Gdm-Induced Macrosomia is Reversed by Cav-1 *Via* AMPK-Mediated Fatty Acid Transport and GLUT1-Mediated Glucose Transport in Placenta. PloS One (2017) 12(1):e0170490. 10.1371/journal.pone.0170490 28125642 PMC5268469

[23] Chadt A Al-Hasani H. Glucose Transporters in Adipose Tissue, Liver, and Skeletal Muscle in Metabolic Health and Disease. Pflugers Arch (2020) 472(9):1273–98. 10.1007/s00424-020-02417-x PMC7462924 32591906

[24] Guo L He B Fang P Bo P Zhang Z. Activation of Central Galanin Receptor 2 Mitigated Insulin Resistance in Adipocytes of Diabetic Rats. J Endocrinol Invest (2021) 44(3):515–22. 10.1007/s40618-020-01336-z 32588381

[25] Colomiere M Permezel M Riley C Desoye G Lappas M. Defective Insulin Signaling in Placenta From Pregnancies Complicated by Gestational Diabetes Mellitus. Eur J Endocrinol (2009) 160(4):567–78. 10.1530/EJE-09-0031 19179458

[26] Peng X Su H Liang D Li J Ting WJ Liao SC. Ramipril and Resveratrol Co-Treatment Attenuates RhoA/ROCK Pathway-Regulated Early-Stage Diabetic Nephropathy-Associated Glomerulosclerosis in Streptozotocin-Induced Diabetic Rats. Environ Toxicol (2019) 34(7):861–8. 10.1002/tox.22758 31062909

[27] Nishiumi S Yoshida M Azuma T Yoshida K Ashida H. 2,3,7,8-Tetrachlorodibenzo-P-Dioxin Impairs an Insulin Signaling Pathway Through the Induction of Tumor Necrosis Factor-Alpha in Adipocytes. Toxicol Sci (2010) 115(2):482–91. 10.1093/toxsci/kfq052 20181658

[28] Bai Y Du Q Zhang L Li L Tang L Zhang W. Fasudil Alleviated Insulin Resistance Through Promotion of Proliferation, Attenuation of Cell Apoptosis and Inflammation and Regulation of RhoA/Rho Kinase/Insulin/Nuclear Factor-kappaB Signaling Pathway in HTR-8/SVneo Cells. J Pharm Pharmacol (2021). 10.1093/jpp/rgab033 33779714

